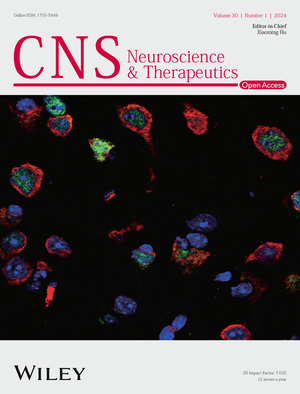# Additional Cover

**DOI:** 10.1111/cns.14626

**Published:** 2024-01-28

## Abstract

The cover image is based on the Research Article *RIPK3 activation promotes DAXX‐dependent neuronal necroptosis after intracerebral hemorrhage in mice* by Qingqing Bai et al., https://doi.org/10.1111/cns.14397.